# Supplementary material for: The Impact of Psilocybin on High Glucose/Lipid-Induced Changes in INS-1 Cell Viability and Dedifferentiation
Source: Genes (Basel). 2024 Jan 29;15(2):183. doi: 10.3390/genes15020183 (PMC10888174; doi:10.3390/genes15020183)
Supplement: Supplementary file 1 [file genes-15-00183-s001.zip › genes-2810770-supplementary.pdf]

Supplementary materials

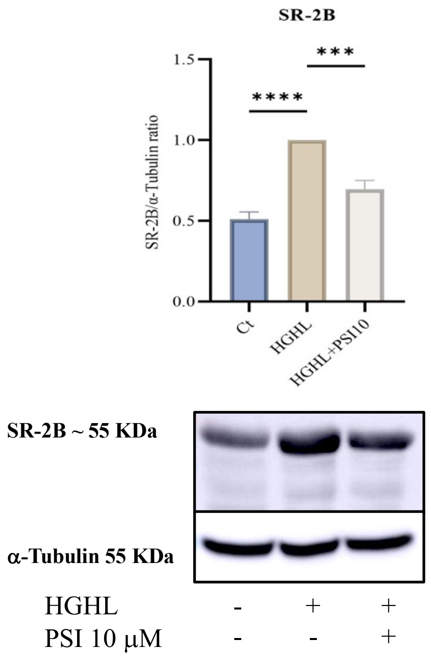

Figure S1. The immunoblot analysis of SR-2B in HG-HL-challenged β-cells in response to 10 μ psilocybin. Psilocybin administration reduced the elevated level of SR-2B in HG-HL-induced INS-1 cells. The results are depicted as the mean value with standard deviation, based on three measurements (N=3). Abbreviations used are Ct (Control), PSI (Psilocybin), and HG-HL (High glucose + High lipid). Significance is denoted by asterisks, with three implying $p<0.001$ and four indicating $p<0.0001$.

Table S1. Information of the primary antibodies

Primary antibody	Manufacturer	Cat #	Dilution
β-actin	Santa Cruz Biotechnology	sc-47778	1:200
Phospho-STAT3(Tyr-705)	Santa Cruz Biotechnology	sc-8059	1:200
STAT3	Santa Cruz Biotechnology	sc-8019	1:200
α-Tubulin	Santa Cruz Biotechnology	sc-8035	1:200
Caspase-3	Cell Signaling Technology	9662s	1:200
Caspase-7	Cell Signaling Technology	12827	1:1000
Bax	Cell Signaling Technology	2772s	1:1000
PDX-1	Cell Signaling Technology	D59H3	1:1000
FOXO1	Cell Signaling Technology	2880S	1:1000
Phospho-FOXO1 (Ser256)	Cell Signaling Technology	9461S	1:1000
β-actin	Abcam	ab8227	1:2000
Phospho-STAT1(S727)	Abcam	ab109461	1:1000
Caspase-9	Santa Cruz Biotechnology	sc-56076	1:100
Cleaved PARP (Asp214)	Cell Signaling Technology	9545	1:200
Bim	Cell Signaling Technology	C34C5	1:1000
Bcl-2	Santa Cruz Biotechnology	sc-7382	1:200
TXNIP	Cell Signaling Technology	14715	1:1000

Table S2: The sequences of primers used for q-RT-PCR

Rat-Ins1	Forward	GGGAACGTGGTTTCTTCTACA
Rat-Ins1	Reverse	CAGTGCCAAGGTCTGAAGAT
Rat-NEUROD1	Forward	GAACACGAGGCAGACAAGAA
Rat-NEUROD1	Reverse	TCATCTTCATCCTCCTCCTCTC
Rat-MafA	Forward	GGTCATCCGACTGAAACAGAA
Rat-MafA	Reverse	CTTCTCGCTCTCCAGAATGTG
Rat-Ins2	Forward	GGGAGCGTGGATTCTTCTACA
Rat-Ins2	Reverse	AGTGCCAAGGTCTGAAGGT
Rat-Slc2A2	Forward	CATAGTCACACCAGCACATACG
Rat-Slc2A2	Reverse	ACAGACAGAGACCAGAGCATAG
Rat-PDX1	Forward	CCCTTTCCCGTGGATGAAATC
Rat-PDX1	Reverse	GCTGTACGGGTCCTCTTATTCT
Rat-FOXO1	Forward	TCTACGAGTGGATGGTGAAGAG
Rat-FOXO1	Reverse	GGACAGATTGTGGCGAATTGA
Rat-Tubulin	Forward	TGACCCTCGCCATGGTAAATA
Rat-Tubulin	Reverse	GATGGTACGCTTGGTCTTGATG
Rat-Nanog	Forward	GCAGCTATTCTCAGGGCTATCT
Rat-Nanog	Reverse	TGGTCCAGGTCTGGTTGTT
Rat-Pou5f1	Forward	CCAAGCTGCTGAAACAGAAGAG
Rat-Pou5f1	Reverse	GTTGTCTGGCTGAACACCTTTC
